# Photosynthetic characteristics of the subtending leaf of cotton boll at different fruiting branch nodes and their relationships with lint yield and fiber quality

**DOI:** 10.3389/fpls.2015.00747

**Published:** 2015-09-17

**Authors:** Jingran Liu, Yali Meng, Fengjuan Lv, Ji Chen, Yina Ma, Youhua Wang, Binglin Chen, Lei Zhang, Zhiguo Zhou

**Affiliations:** ^1^Key Laboratory of Crop Physiology and Ecology in Southern China, Ministry of Agriculture, Nanjing Agricultural UniversityNanjing, China; ^2^State Key Laboratory of Cotton Biology, Institute of Cotton Research, Chinese Academy of Agricultural SciencesAnyang, China

**Keywords:** cotton (*Gossypium hirsutum* L.), subtending leaf of cotton boll, fruiting branch nodes, chlorophyll, photosynthesis, yield and quality

## Abstract

To investigate photosynthetic characteristics of the subtending leaf at the 2–3rd and 10–11th fruiting branch (FBN, FB_2–3_, and FB_10–11_), and their relationship with cotton yield and quality, field experiments were conducted using two cotton cultivars, Kemian 1 and Sumian 15. The results showed that with FBN increasing, chlorophyll (Chl) components, *Pn* and non-photochemical quenching (NPQ) in the subtending leaf significantly declined, while soluble sugar, amino acid and their ratio (*C*_SS_/*C*_AA_) as well as *F*_v_/*F*_m_ increased. These results indicated that (1) non-radiative dissipation of excess light energy at FB_2–3_ was reduced to improve solar energy utilization efficiency to compensate for lower *Pn*, (2) higher NPQ at FB_10−11_ played a role in leaf photo-damage avoidance, (3) boll weight was related to the *C*_SS_/*C*_AA_ ratio rather than carbohydrates content alone, (4) with FBN increasing, lint biomass and lint/seed ratio increased significantly, but lint yield decreased due to lower relative amount of bolls, and (5) the decreases in *Pn*, sucrose content and *C*_*SS*_/*C*_*AA*_ in the subtending leaf at FB_2–3_ resulted in lower boll weight and fiber strength.

## Introduction

Photosynthesis is an integrated and regulated process highly sensitive to any change in environmental conditions, because it needs to balance the light energy absorbed by the photosystems with the energy consumed by the metabolic sinks of a plant (Ensminger et al., 2006). The photosynthesis of canopy is associated positively with chlorophyll (Chl) content which decreases at late season (Wells, 2001). In addition, environmental stresses decrease the performance of the photosystem (PS), especially that of PS II; thus, Chl fluorescence is considered a valuable tool to detect the influence of stress factors on plant photosynthesis (Singh et al., 2013). The absorbed light energy that exceeds the photochemical processes of CO_2_ fixation can be either dissipated as heat or re-emitted as Chl fluorescence (Maxwell and Johnson, 2000). Non-photochemical quenching (NPQ) plays an important role in the non-radiative dissipation of excess light energy (Zhao et al., 2007), and maximal photochemical efficiency of PS II (*F*_v_/*F*_m_) has been used to indicate the potential quantum efficiency of PS II (Lichtenthaler and Babani, 2004) and to monitor plants' responses to environmental stress (Ramalho et al., 2003). Previous studies have shown that higher NPQ in the leaves resulted in a stronger deep-oxidation of the large xanthophyll cycle pool (Demmig-Adams and Adams, 1996; Anderson, 1999), and could act as a major defense mechanism to reduce the formation of reactive oxygen species in PS II, and subsequently to avoid or mitigate photo-damage to leaves (Liu et al., 2001; Zhao et al., 2007).

Chl is essential for converting light energy into stored chemical energy and is an important indicator for evaluating leaf photosynthesis. A decrease in Chl content might also result in an increase of the minimum fluorescence yield (Fo) by lowering the re-absorption and increasing the emission of fluorescence light (Keutgen and Chen, 2001). During the late growth period of wheat, Chl b was found to be more important for improving photosynthesis (Wang, 2007). In addition, Rubisco activity diminished in accordance with Chl degradation and photosynthesis changes (Crafts-Brandner et al., 1990). Since carbon metabolism is regulated to efficiently utilize limited Pi resources, carbon is partitioned into free amino acids for maintaining the carbon skeletons (Paul and Pellny, 2003), which also affects crop quality (Liu et al., 2007; Halford, 2010). The ratio of leaf carbon to nitrogen has been used to calculate the allocation of photosynthetic electrons and the assimilation of nitrogen necessary for amino acid formation (Lewis et al., 2000). Moreover, nitrogen assimilation requires energy and carbon skeletons; these resources are diverted from carbohydrate metabolism, thus explaining the decrease in carbohydrate content when nitrogen assimilation is stimulated (Invers et al., 2004).

In cotton, approximately 60–87% of the carbon in a mature boll is derived from CO_2_ assimilation during boll development period; during this process, the subtending leaf is the most important contributor to biomass accumulation in seed cotton (Ashley, 1972; Constable and Rawson, 1980; Wullschleger and Oosterhuis, 1990). Bolls and their subtending leaves are the sinks and sources for photosynthates. Their relationships could reflect the coordination of the vegetative and reproductive growths of cotton and affect cotton yield and quality (Xie et al., 2003). Previous studies have shown that an earlier sink formation ability and stronger reproductive growth potential are important characteristics of high-yield cotton varieties (Pace et al., 1999). High grain weight and yield can be obtained by ensuring not only stronger photosynthesis in the functional leaf (source activity) but also effective distribution of photosynthates to the reproductive organs (Richards, 2000; Wang, 2007). It was suggested that the partitioning of carbon and nitrogen to the reproductive meristem needs to be increased to ensure greater grain number and size (Richards, 2000).

Improving cotton yield requires the investigation of suitable leaf area index (Wang et al., 2002) and canopy-apparent photosynthesis during boll development, and improving Chl content and photosynthesis rate (Wang et al., 2002; Pettigrew et al., 2007). Cotton boll biomass and fiber quality are known to be influenced by environmental conditions (Gormus and Yucel, 2002; Boquet and Clawson, 2009). Therefore, the effects of environmental conditions such as temperature and light on cotton bolls and their subtending leaves can be avoided by selecting different fruiting branch nodes (FBN) at the same flowering date (Wang et al., 2006). In the previous study, FBN was used to characterize the physiological age of cotton (Liu et al., 2014). It was found that at the same flowering date, for physiologically old cotton, the sucrose phosphate synthase activity and sucrose content in the subtending leaf were higher, and the sucrose phosphate synthase activity, sucrose content and sucrose transformation rate in fiber was also higher, compared to physiologically young cotton. All these indicated that more carbon in the subtending leaf was transported into the boll during boll development, which was in favor of cellulose accumulation in fiber, thereby increasing boll weight and fiber strength (Shu, 2009; Zhao et al., 2011; Liu et al., 2014). For *Arabidopsis thaliana*, the contents of Chl and soluble protein were observed to be slightly higher in the leaves of an 8-week-old plant than in a 6-week-old plant, although the two groups of leaves were at the same stage of senescence (Zentgraf et al., 2004). However, little is known about the relationship of photosynthetic characteristics with cotton yield and fiber quality, except Zhao and Oosterhuis (2000) found that an increase in FBN within a plant remarkably decreased leaf biomass, which was related with leaf photosynthesis (Zhao and Oosterhuis, 2000).

In this study, we used two different cotton cultivars that were planted at different dates to provide different FBN, and investigated changes in photosynthetic characteristics of the subtending leaf at different FBN and their relationships with lint yield and fiber quality with the aim to provide a theoretical basis for cultivating varieties with high yield and fine quality.

## Materials and methods

### Experimental design

Field experiments were conducted at Jiangsu Academy of Agricultural Sciences in 2009 and at Pailou Experimental Station in 2010 and 2011, Nanjing, Jiangsu, China (118° 50′ E, 32° 02′ N). The soils at the experimental site were clay, mixed, thermic, Typical Udalfs, Alfisols. Before sowing cotton, the soil profiles at 20 cm depth during 2009, 2010 and 2011 were 20.1, 18.3 and 18.5 g kg^−1^ organic matter; 1.2, 1.1 and 1.0 g kg^−1^ total N; 110, 65 and 81 mg kg^−1^ mineral N (${\text{NH}}_{4}^{+}$-N and ${\text{NO}}_{3}^{-}$-N); 20, 18 and 19 mg kg^−1^ Olsen P; and 111, 102 and 111 mg kg^−1^ exchangeable K (NH_4_OAc-K), respectively. Two cotton cultivars widely grown in the Yangtze River Valley of China—Kemian 1 and Sumian 15—were planted in the field on 25-Apr and 25-May each year. Cotton seeds were initially planted in nutrition bowls in a nursery bed, and thus the seedlings at the three–leaf stage were transplanted into the field. Three replications were randomly assigned for each treatment. Furrow irrigation was applied as needed during each season to minimize moisture stress.

### Sampling and processing

White flowers on the first position of the 10–11th fruiting branches of plants planted on 25-Apr (FB_10–11_) and the 2–3rd fruiting branches of plants planted on 25-May (FB_2–3_) of all plants were simultaneously labeled with small plastic tags to ensure uniform metabolic and developmental ages of flowers. The subtending leaves under the labeled flowers were collected at 09:00–10:00 a.m. once every 7 days from 10 days post-anthesis (DPA) until the boll opening date; they were immediately transported in an ice box to the laboratory for analysis. The leaves were washed with distilled water and cut along the main vein into two halves. One half was placed immediately in liquid nitrogen for subsequent Chl measurement, whereas the other was dried at 105°C for 30 min and then at 70°C to constant weight and used for measuring soluble sugar and amino acid contents.

### Photosynthesis and Chl fluorescence in the subtending leaf

Net photosynthetic rate (*Pn*) of the labeled subtending leaves oriented perpendicular to the sun at 17 and 31 DPA in 2009 and at 17, 31, and 45 DPA in 2010 and 2011 was measured by using the Li-6400 portable photosynthesis system (Li-COR Inc., NE, USA) under the following conditions: 1500 μmol m^−2^ s^−1^ light intensity, (65 ± 5)% relative humidity, (32 ± 2)°C leaf temperature and 380 μmol mol^−1^ CO_2_ during 9:30–11:00 a.m.

In parallel with the photosynthesis measurement, Chl fluorescence was measured using the same leaves by using PAM 2000 (Germany). A dark leaf clip was placed on each leaf following the photosynthesis measurement and the leaves were allowed to adapt to the dark for at least 30 min before the measurement of the minimum and maximum Chl fluorescence (*F*_0_ and *F*_m_, respectively). The maximum and steady-state Chl fluorescence in the light-adapted leaf ($F_{\text{m}}^{\prime }$ and *F*_s_, respectively) were also measured. These data were used to calculate the maximal and actual photochemical efficiency of PS II by using the following equations; *F*_v_/*F*_m_ = (*F*_m_ − *F*_0_)/*F*_m_ and ϕ_PS II_ = ($F_{\text{m}}^{\prime }$ − *F*_s_)/$F_{\text{m}}^{\prime }$, respectively.

### Pigment content, soluble sugar content and amino acid content in the subtending leaf

Chl was extracted from fresh leaf tissue treated with 25 mL of ethanol/acetone (1/1, v/v) for 24 h in the dark. Chl components were then quantified spectrophotometrically at 663 and 645 nm (Moran, 1982).

Dried leaf tissues (0.1 g) were placed in a 10 mL centrifuge tube and mixed with 5 mL of 80% ethanol. The mixture was incubated in a water bath with shaking at 80°C for 30 min, and centrifuged at 4000 rpm for 5 min to collect the supernatants. The pellets were extracted two more times with 80% ethanol, and all the supernatants were combined and diluted to 25 mL with 80% ethanol, mixed, and stored at −20°C for measuring the soluble sugar and amino acid contents by using the anthrone method (Seifter et al., 1950), and acid ninhydrin reagent (Rosen, 1957) method, respectively.

### Lint yield and fiber quality

Thirty cotton plants in each treatment were randomly selected every year for measuring the number of bolls. Carpel, seed and lint biomass per boll (g boll^−1^) and their distribution rates (%) as well as boll weight (g) were obtained from the 30 bolls that were hand-collected from plants at 0.5 m of the outer two rows. Fiber quality characteristics for each treatment were determined using a high-volume instrument (HVI) at the Cotton Quality Supervision, Inspection and Testing Center of the Ministry of Agriculture, China.

### Data analysis

All data were subjected to analysis of variance using SPSS (ver. 17.0; SPSS, Chicago, IL, USA) software, and the differences between means were identified using least significant difference (LSD) method (*P* < 0.05). In addition, the relative amount of bolls was calculated as 100% × (the number of bolls on FB_1−5_, FB_10−15_, respectively/total number of bolls per plant).

## Results

### Meteorological data during boll development

**Figure 1:**
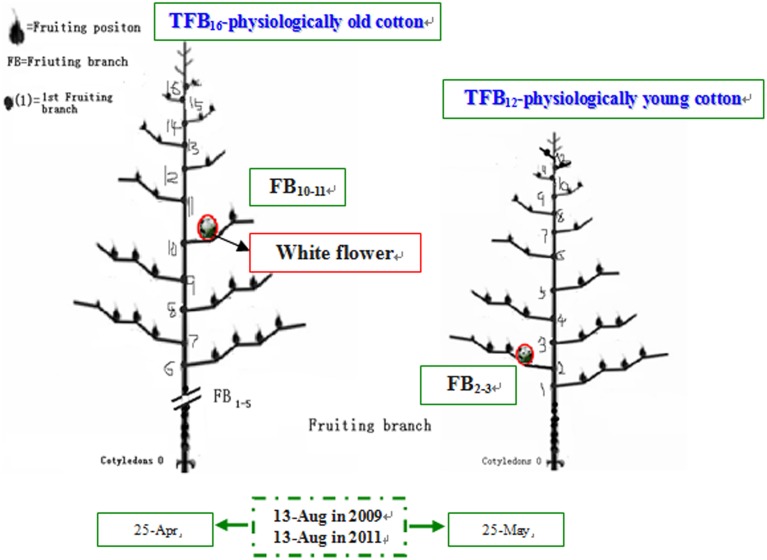
**Schematic diagram of cotton physiological age in 2009 and 2011 adapted from Liu et al. (2014)**.

Meteorological data during boll development was obtained from Nanjing Meteorological Station. The growth and development conditions of bolls and their subtending leaves at different FBN (FB_10–11_ and FB_2–3_) were similar at the same flowering time for cotton plants (Table 1). Cotton plants at FB_2–3_ were characterized as physiologically young cotton (TFB_12_) and those at FB_10–11_ as physiologically old cotton (TFB_16_; Figure 1) (Liu et al., 2014).

**Table 1:** **Meteorological data and fruiting branch nodes during boll development at different planting dates during 2009 to 2011**.

**Years**	**Flowering dates**	**Boll Period (d)**	**MDT (°C)**	**MDT_max_(°C)**	**MDT_min_(°C)**	**Total sunshine hours (h)**	**Mean daily solar radiation (MJ·m^−2^)**	**Fruiting branch nodes (FBN)**	
								**25-Apr**	**25-May**
2009	13-Aug	55	24.8	28.8	21.9	274.9	14.4	10–11th (FB_10–11_)	_2–3_rd (FB_2–3_)
2010	10-Aug	46	27.3	31.6	24.3	276.8	17.1	10–11th	_2–3_rd
2011	13-Aug	55	23.7	27.6	20.8	254.4	13.7	10–11th	_2–3_rd

MDT, MDT_max_, and MDT_min_ were mean daily temperature, mean daily maximum temperature, mean daily minimum temperature, respectively.

### Source (subtending leaf) strength of cotton plants at different fruiting branch nodes (FBN)

#### Photosynthetic pigments in the subtending leaf

**Figure 2:**
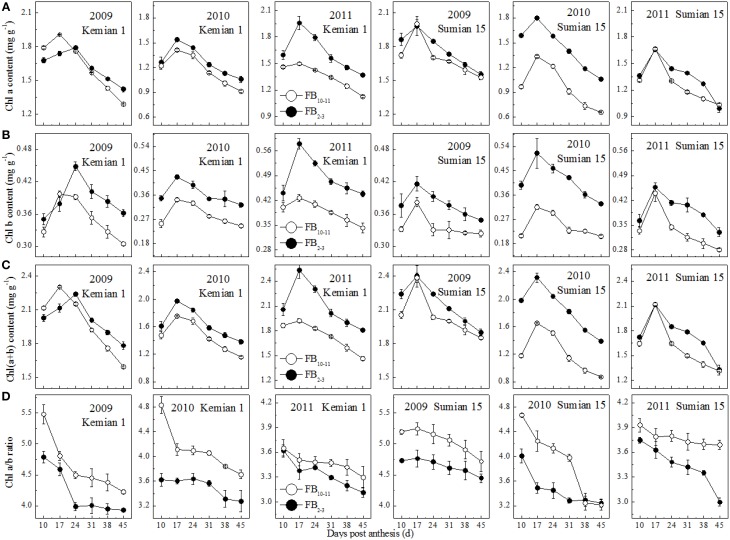
**Changes in chlorophyll components of the subtending leaf at different fruiting branch nodes (FBN)**.

The contents of Chl a, Chl b and Chl(a+b) of the two cultivars decreased significantly from 17 DPA to boll opening during the 3 years, and decreased with FBN increasing (Figure 2). Compared with those at FB_2–3_, the Chl a and b contents at FB_10–11_ increased by 9.7–12.9% and 15.5–21.5% for Kemian 1 and by 6.4–32.4% and 14.4–40.5% for Sumian 15, respectively. Moreover, the degree of decrease in Chl(a+b) content at FB_10–11_ was similar to that of decrease in Chl a content. These results indicated that, of the three photosynthetic pigments, Chl b was the most sensitive to FBN.

The Chl a/b ratio in the subtending leaf declined with DPA increasing, and increased with FBN increasing (Figure 2D), indicating that Chl a content at FB_10–11_ decreased more rapidly than Chl b content. In addition, the difference in the Chl a/b ratio between FB_10–11_ and FB_2–3_ was the highest in 2010.

#### Pn and Chl fluorescence in the subtending leaf

*Pn*, ϕ_PS II_, and *F*_v_/*F*_m_ reduced with DPA increasing, and increased with FBN increasing (Tables 2, 3). Compared with FB_10–11_, *Pn* and ϕ_PS II_ at FB_2–3_ decreased by 12.0–28.8% and 3.1–3.2% for Kemian 1 and by 16.0–35.9% and 4.0–8.0% for Sumian 15, respectively. Moreover, NPQ had an increasing trend with DPA and FBN increasing. However, the *F*_v_/*F*_m_ at FB_10–11_ and FB_2–3_ was not significantly different between the two cultivars.

**Table 2:** **Changes in net photosynthetic rate (*Pn*, μmol CO_2_ m^−2^ s^−1^) of the subtending leaf during cotton boll development at different fruiting branch nodes (FBN) during 2009 to 2011**.

**FBN**	**Days post-anthesis (d) 2009**			**Days post-anthesis (d) 2010**			**Days post-anthesis (d) 2011**		
	**17**	**31**	**45**	**17**	**31**	**45**	**17**	**31**	**45**
**KEMIAN 1**									
FB_10–11_	22.0 a	18.2 a	10.1 a	22.7 a	20.2 a	10.3 a	23.7 a	16.8 a	9.4 a
FB_2–3_	18.8 b	16.1 b	9.3 a	14.8 b	13.3 b	9.8 b	20.8 b	12.9 b	9.2 b
**SUMIAN 15**									
FB_10–11_	21.5 a	18.4 a	10.5 a	21.9 a	19.6 a	10.5 a	22.9 a	16.8 a	9.1 a
FB_2–3_	18.8 b	14.5 b	9.0 b	14.1 b	10.8 b	8.4 b	19.1 b	12.5 b	7.5 b

Values followed by a different letter within the same column are significantly different at P = 0.05 probability level.

**Table 3:** **Actual PSII efficiency (ϕ_PS II_), maximal efficiency of PSII photochemistry (*F*_v_/*F*_m_) and non-photochemical quenching (NPQ) in the subtending leaf during cotton boll development at different fruiting branch nodes (FBN) during 2009 and 2011**.

**Cultivars**	**FBN**	**Days post-anthesis (d)**					
		**2009**			**2011**		
		**17**	**31**	**45**	**17**	**31**	**45**
**ϕ_PS II_**							
Kemian 1	FB_10–11_	0.64 a	0.57 a	0.54 a	0.69 b	0.65 a	0.52 a
	FB_2–3_	0.68 a	0.62 a	0.56 a	0.73 a	0.66 a	0.53 a
Sumian 15	FB_10–11_	0.52 b	0.53 b	0.51 a	0.68 b	0.61 a	0.45 a
	FB_2–3_	0.67 a	0.64 a	0.52 a	0.71 a	0.63 a	0.47 a
***F*_v_/*F*_m_**							
Kemian 1	FB_10–11_	0.82 a	0.78 a	0.71 a	0.76 a	0.75 a	0.65 a
	FB_2–3_	0.84 a	0.81 a	0.72 a	0.79 a	0.76 a	0.63 a
Sumian 15	FB_10–11_	0.80 a	0.73 a	0.69 a	0.74 a	0.72 a	0.63 a
	FB_2–3_	0.81 a	0.74 a	0.71 a	0.77 a	0.73 a	0.67 a
**NPQ**							
Kemian 1	FB_10–11_	0.81 a	0.84 a	0.91 a	0.75 a	0.86 a	0.89 a
	FB_2–3_	0.78 a	0.82 a	0.90 a	0.73 a	0.85 a	0.86 b
Sumian 15	FB_10–11_	0.74 a	0.81 a	0.89 a	0.77 a	0.83 a	0.87 a
	FB_2–3_	0.73 a	0.80 a	0.86 b	0.73 b	0.82 a	0.84 b

Values followed by a different letter within the same column are significantly different at P = 0.05 probability level.

#### Soluble sugar and amino acid contents in the subtending leaf

**Figure 3:**
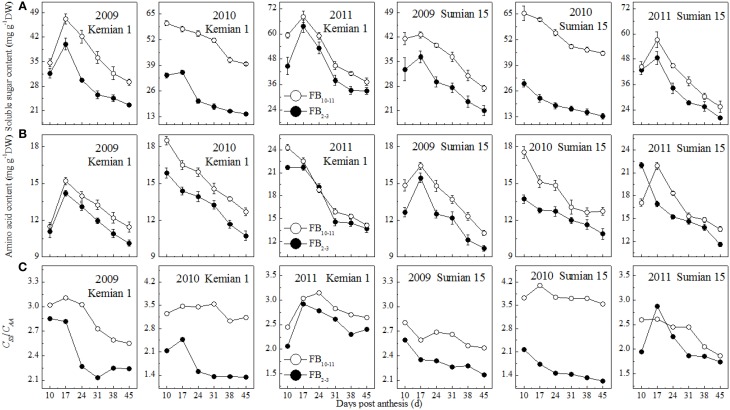
**Changes in soluble sugar content, amino acid content and *C*_*SS*_/*C*_*AA*_ of the subtending leaf at different fruiting branch nodes (FBN)**.

The contents of soluble sugars and amino acids in the subtending leaf reduced with DPA increasing, and increased with FBN increasing (Figures 3A,B). Compared with those at FB_2–3_, soluble sugar and amino acid contents at FB_10–11_ were increased by 14.3–54.6% and 5.1–13.2% for Kemian 1 and by 17.1–64.0% and 6.8–14.2% for Sumian 15, respectively. The difference in soluble sugar content between FB_2–3_ and FB_10–11_ was the highest in 2010 and even higher than that of the amino acid content.

The ratio of soluble sugar to amino acid content (*C*_*SS*_/*C*_*AA*_ ratio) decreased with DPA and increased with FBN increasing (Figure 3C), suggesting that soluble sugar content decreased more rapidly than amino acid content with DPA increasing, but increased more rapidly than amino acid with FBN increasing. In addition, the *C*_*SS*_/*C*_*AA*_ ratio was higher for Kemian 1 than for Sumian 15, but declined more rapidly, because of the higher soluble sugar content (Figure 3A) and gradual decrease in amino acid content (Figure 3B).

### Sink strength (cotton biomass, lint yield, and fiber quality characteristics) at different FBN

The biomass of carpel, seed and lint increased with FBN increasing (Table 4). Only seed and lint biomasses for Sumian 15 were significantly affected by FBN. In addition, lint distribution rate and lint/seed ratio increased significantly with FBN increasing (*P* < 0.05). However, there were no significant differences in distribution rates of carpel and seed between FB_10–11_ and FB_2–3_.

**Table 4:** **Changes of the biomass and distribution of boll components at different fruiting branch nodes (FBN) during 2009 to 2011**.

**FBN**	**Boll biomass (g boll**^**−1**^**)**						**Distribution rate (%)**						**Lint/seed ratio**	
	**Carpel**		**Seed**		**Lint**		**Carpel**		**Seed**		**Lint**			
	**K**	**S**	**K**	**S**	**K**	**S**	**K**	**S**	**K**	**S**	**K**	**S**	**K**	**S**
**2009**														
FB_10–11_	2.1 a	2.0 a	3.3 a	3.5 a	2.3 a	2.4 a	27.0 a	25.0 a	43.1 a	44.9 a	29.9 a	30.1 a	0.69 a	0.67 a
FB_2–3_	2.0 a	1.8 a	3.2 a	3.3 b	2.2 a	2.1 b	26.8 a	24.9 a	43.8 a	45.8 a	29.4 b	29.3 b	0.67 b	0.64 b
**2010**														
FB_10–11_	1.8 a	1.6 a	3.3 a	3.4 a	2.4 a	2.3 a	23.5 a	21.2 a	44.3 a	46.9 a	32.2 a	31.8 a	0.73 a	0.68 a
FB_2–3_	1.7 a	1.5 a	3.2 a	3.2 a	2.3 a	2.1 b	23.8 a	21.9 a	44.4 a	47.1 a	31.9 a	31.0 b	0.72 a	0.66 a
**2011**														
FB_10–11_	1.7 a	1.6 a	3.5 a	3.3 a	2.2 a	2.2 a	22.6 a	22.5 a	47.3 a	46.7 a	30.2 a	30.8 a	0.64 a	0.66 a
FB_2–3_	1.6 a	1.5 a	3.4 a	3.1 b	2.1 a	1.9 b	23.0 a	22.9 a	47.6 a	47.2 a	29.4 b	30.0 b	0.62 b	0.64 b

K and S Kemian 1 and Sumian 15.

Values followed by a different letter within the same column are significantly different at P = 0.05 probability level.

Carpel distribution rate = Carpel biomass/(carpel + seed + lint) biomass × 100; Seed distribution rate = Seed biomass/(carpel + seed +lint) biomass × 100; Lint distribution rate = Lint biomass/(carpel + seed +lint) biomass × 100; Lint/seed ratio = Lint/seed biomass.

For both the cultivars, the relative amount of bolls reduced, and boll weight increased significantly with FBN increasing (*P* < 0.05; Table 5). Compared with that at FB_2–3_, lint yield at FB_10–11_ was higher by 33.4–36.6% and 34.2–41.8% for Kemian 1 and Sumian 15, respectively.

**Table 5:** **Changes in the relative amount of bolls, boll weight and lint yield of the two cultivars Kemian 1 and Sumian 15 at different fruiting branch nodes (FBN) during 2009 to 2011**.

**FBN**	**Relative amount of bolls (%)**			**Boll weight (g boll**^**−1**^**)**			**Lint yield (kg ha**^**−1**^**)**		
	**2009**	**2010**	**2011**	**2009**	**2010**	**2011**	**2009**	**2010**	**2011**
**KEMIAN 1**									
FB_10–11_	25.4 b	26.8 b	22.3 b	5.7 a	5.7 a	5.7 a	404 b	468 b	393 b
FB_2–3_	39.5 a	38.4 a	34.4 a	5.4 a	5.5 a	5.5 a	541 a	640 a	524 a
**SUMIAN 15**									
FB_10–11_	22.4 b	25.3 b	20.7 b	5.9 a	5.8 a	5.5 a	349 b	441 b	341 b
FB_2–3_	38.7 a	41.6 a	37.2 a	5.5 b	5.3 b	5.0 b	481 a	626 a	458 a

Relative amount of bolls was calculated as 100% × (the number of bolls on FB_1−5_, FB_10−15_, respectively/total number of bolls per plant).

Values followed by a different letter within the same column are significantly different at P = 0.05 probability level.

Fiber strength, but not fiber length, elongation and uniformity index, was significantly different between FB_10–11_ and FB_2–3_. Fiber strength increased by 2.4–7.1% and 5.7–7.9% with FBN increasing for Kemian 1 and Sumian 15, respectively (Table 6). In addition, micronaire value increased significantly with FBN increasing, but was still within the state standard scale (Class A as the best range, 3.7–4.2; Class B as the standard level, 3.5–3.6 or 4.3–4.9).

**Table 6:** **Changes in fiber quality characteristics and the interactions between planting dates and cultivars to fiber quality at different fruiting branch nodes (FBN) during 2009 to 2011**.

**FBN**	**Fiber length (mm)**			**Elongation (%)**			**Fiber strength (cN tex**^**−1**^**)**			**Micronaire**			**Uniformity index (%)**		
	**2009**	**2010**	**2011**	**2009**	**2010**	**2011**	**2009**	**2010**	**2011**	**2009**	**2010**	**2011**	**2009**	**2010**	**2011**
**KEMIAN 1**															
FB_10–11_	29.1 a	29.1 a	29.8 a	6.7 a	6.3 a	6.5 a	29.7 a	31.9 a	29.7 a	4.9 a	5.4 a	4.9 a	87 a	85 a	84 a
FB_2–3_	29.5 a	29.6 a	29.8 a	6.7 a	6.4 a	6.6 a	29.0 a	29.8 b	28.5 b	4.3 b	4.6 b	4.7 b	86 a	85 a	84 a
**SUMIAN 15**															
FB_10–11_	29.5 a	28.4 a	29.4 a	6.8 a	6.1 a	6.3 a	29.7 a	27.9 a	28.6 a	4.4 a	5.4 a	4.5 a	86 a	82 a	84 a
FB_2–3_	29.6 a	29.1 a	29.4 a	6.9 a	6.3 a	6.3 a	28.1 b	26.4 b	26.5 b	3.9 b	4.4 b	4.3 b	86 a	84 a	83 a

Values followed by a different letter within the same column are significantly different at P = 0.05 probability level.

### Correlation of sink (boll) strength with source (subtending leaf) strength IN cotton

Lint biomass was significantly negatively correlated with Chl b content and significantly positively correlated with *Pn* (*P* < 0.05; Table 7). Lint distribution rate was significantly negatively correlated with Chl components. However, lint/seed ratio was significantly negatively correlated only with Chl b content (*P* < 0.05).

**Table 7:** **Regression coefficients between source indices and sink indices at different fruiting branch nodes (FBN) during 2009 to 2011**.

**Correlation with**	**Chl a**	**Chl b**	**Chl(a+b)**	***Pn***	***C_*SS*_***	***C_*SS*_*/*C_*AA*_***
Lint biomass	ns	−0.668^*^	ns	0.659^*^	ns	ns
Lint distribution rate	−0.828^**^	−0.632^*^	−0.847^**^	ns	ns	Ns
Lint/seed ratio	ns	−0.665^*^	ns	ns	ns	Ns
Relative amount of bolls	ns	ns	ns	−0.866^**^	−0.742^**^	−0.649^*^
Boll weight	ns	ns	ns	0.774^**^	ns	0.670^*^
Lint yield	ns	ns	ns	−0.769^**^	−0.602^*^	Ns
Strength	ns	ns	ns	0.655^*^	ns	Ns
Micronaire	−0.780^**^	ns	−0.782^**^	0.628^*^	0.762^**^	0.748^**^

n = 12, $R_{0.05}^{2}=0.576$, $R_{0.01}^{2}=0.707$.

ns, not significant at P < 0.05;

* P < 0.05;

** P < 0.01.

The relative amount of bolls was significantly negatively correlated with *Pn*, soluble sugar and *C*_*SS*_/*C*_*AA*_ (*P* < 0.05). Boll weight was significantly negatively correlated with *Pn* and *C*_*SS*_/*C*_*AA*_. Lint yield was significantly negatively correlated with soluble sugar and *Pn*.

Fiber strength was significantly positively correlated with *Pn*. Micronaire value was significantly negatively correlated with Chl a and Chl(a+b), and positively correlated with *Pn*, soluble sugar content, and *C*_*SS*_/*C*_*AA*_ (*P* < 0.05).

## Discussion

In our previous study (Liu et al., 2014), we showed that the characteristics of subtending leaves with the same flowering time but at different FBN of plants seeded at different dates could more accurately reflect the growth conditions of bolls and their subtending leaves. Boll weight and fiber quality at different FBN at the same planting date were found to be significantly different (Zhao and Oosterhuis, 2000; Davidonis et al., 2004). In cotton, about 60–87% of carbon in a mature boll comes from its subtending leaf (Ashley, 1972; Constable and Rawson, 1980; Wullschleger and Oosterhuis, 1990). Therefore, the subtending leaf plays a crucial role in improving cotton yield, particularly boll weight during cotton development.

Chl a, the reaction center pigment, is able to convert light energy into electrical energy, and Chl b plays an essential role in absorbing blue violet light, which is important to improve the light-trapping ability (Wang et al., 2011). In this study, the contents of Chl a, Chl b and Chl(a+b) as well as the Chl a/b ratio in the subtending leaf were higher at FB_2–3_ than at FB_10–11_, suggesting that light absorption was increased and the light energy utilization was improved in the subtending leaf at FB_2–3_, which was consistent with the findings of previous studies performed under shading (Sarijeva et al., 2007). ϕ_PS II_ as the non-cyclic electron transfer efficiency or light energy capture efficiency, could reflect the actual primary light energy conversion efficiency of the PS II reaction center (Li et al., 2010). A significantly higher ϕ_PS II_ value at FB_2–3_ indicated that the subtending leaf could efficiently convert photon energy to chemical energy, which might have led to the lower *Pn*. This phenomenon needs to be further investigated. Previous studies showed that low Chl a/b ratio indicated higher light-harvesting pigment protein content, which has a negative correlation with NPQ (Anderson, 1999). At FB_10–11_, a higher NPQ of the subtending leaf resulted in greater deep oxidation of the large xanthophyll cycle pool (Demmig-Adams and Adams, 1996; Anderson, 1999), and could act as a major defense mechanism to reduce the formation of reactive oxygen species in PS II and subsequently to avoid or mitigate photo-damage to leaves. These results were consistent with those of previous studies (Liu et al., 2001; Zhao et al., 2007). Numerous studies indicated that when the Pi-regeneration capacity is not restricted, Rubisco might be the main limiting factor for *Pn* in plants (Makino and Sage, 2007; Yamori et al., 2009). However, the increase in Rubisco activity did not result in higher *Pn* in the subtending leaf at FB_2–3_ (Liu et al., 2014). This could be because of the Pi regeneration limitation in the subtending leaf during photosynthesis occurred at FB_2–3_. This phenomenon was also observed in rice (*Oryza sativa* L.) (Maruyama et al., 1990).

As carbon metabolism is regulated to efficiently utilize limited Pi resources, carbon is partitioned into free amino acids for carbon skeletons (Paul and Pellny, 2003), which also affects crop quality (Liu et al., 2007; Halford, 2010). At the same flowering date, the contents of soluble sugar and amino acid increased with FBN increasing (Figures 2A,B). Physiologically, as the numbers of fruiting branches and bolls increased rapidly and cotton growth transitioned from vegetative growth to reproductive growth, the physiological age of cotton also increased (Liu et al., 2014) and more carbon in the subtending leaf was stored rather than exported efficiently to the boll. In addition, in *Arabidopsis* soluble protein content in leaves at the same stage of senescence was observed to be slightly higher in 8-week-old plants than in 6-week-old plants (Zentgraf et al., 2004). Because most proteins are made of amino acids, as a consequence, the cause for the higher amino acid content in the subtending leaf of FB_10–11_ might result from blocked protein synthesis or accelerated protein degradation and needs to be further investigated. In this study, boll weight had a significant negative correlation with *C*_*SS*_/*C*_*AA*_ ratio (Table 7) and sucrose content (Liu et al., 2014), but not with soluble sugar and amino acid in the subtending leaf. This could be because soluble sugar in the subtending leaf mainly refers to water-soluble monosaccharides and oligosaccharides such as sucrose and hexose (glucose and fructose), and sucrose in the subtending leaf is the main carbohydrate translocated from source to sink tissues (Farrar et al., 2000). In addition, carbon metabolism is known to be associated with nitrogen metabolism in plants, and sugar content and the ratio of carbon to nitrogen could also be used as an indicator of the status of plant nitrogen nutrition in crops (Lee et al., 1989). These results indicated that the regulation of boll weight might depend on the ratio of carbon to nitrogen instead of carbohydrates alone (Martin et al., 2002; Wingler et al., 2006). Therefore, carbon and nitrogen balance in the subtending leaf is conductive to carbohydrate synthesis and transport, which could provide a theoretical support for improving cotton yield and quality.

The biomass accumulation in boll components is the basis for increasing cotton yield and quality; it is a dynamic process for carbohydrate competition and allocation in the various components of bolls. In this study, higher relative amount of bolls at FB_2–3_ than at FB_10–11_ (Table 6) probably resulted from the reduced the number of bolls and increased source/sink ratio at FB_2–3_ (Liu et al., 2014). Previous studies have shown that higher soluble sugar contents were conductive to improving boll weight (Sun et al., 2004), and micronaire value was linearly associated with the amount of canopy photosynthesis from 15 to 45 DPA (Bauer et al., 2000). In addition, boll weight was significantly negatively correlated with *Pn* and *C*_*SS*_/*C*_*AA*_; the relative amount of bolls and lint yield were negatively correlated with soluble sugar and *Pn*; and fiber strength was significantly positively correlated with *Pn* (*P* < 0.05, Table 7). Therefore, higher lint yield at FB_2–3_ than at FB_10–11_ might be mainly due to higher relative amount of bolls at FB_2–3_. Furthermore, the decreases of *Pn*, sucrose content and *C*_*SS*_/*C*_*AA*_ in the subtending leaf are the main reasons for lower boll weight and fiber strength at FB_2–3_ than at FB_10–11_.

## Conclusions

Our results suggest the following:

1. Higher ϕ_PS II_ value of the subtending leaf at FB_2–3_ led to reduced non-radiative dissipation of excess light energy and improved solar energy utilization efficiency to compensate for the lower *Pn*. In addition, higher NPQ at FB_10–11_ could act as a major defense mechanism in form of heat dissipation to avoid or mitigate photo-damage to leaves.
2. The regulation of boll weight at different FBN depended on the *C*_*SS*_/*C*_*AA*_ ratio in the subtending leaf rather than carbohydrates content alone.
3. Lint yield was higher at FB_2–3_ than at FB_10–11_, mainly because of higher relative amount of bolls.
4. The decrease of *Pn*, sucrose content and *C*_*SS*_/*C*_*AA*_ in the subtending leaf at FB_2–3_ resulted in lower boll weight and fiber strength.

## Author contributions

YLM, YW, BC, and ZZ conceived and designed the experiments. JL, YLM, FL, JC, YNM, and LZ performed the experiments. JL analyzed the data. JL and YLM contributed to reagents/materials/analysis tools. JL wrote the paper.

### Conflict of interest statement

The authors declare that the research was conducted in the absence of any commercial or financial relationships that could be construed as a potential conflict of interest.

## Abbreviations

DPA: days post-anthesis
FBN: fruiting branch nodes
FB_10–11_: the 10–11th fruiting branches
FB_2–3_: the 2–3rd fruiting branches
*F*_v_/*F*_m_: maximal photochemical efficiency of PS II
NPQ: non-photochemical quenching
*Pn*: net photosynthetic rate
ϕ_PS II_: actual PS II efficiency
*C*_SS_/*C*_AA_: ratio the ratio of soluble sugar content to amino acid content.
